# Colonic ischemia possibly due to resuscitative endovascular balloon occlusion of the aorta (REBOA) used to manage amniotic fluid embolism: a case report

**DOI:** 10.1186/s40981-019-0266-6

**Published:** 2019-07-20

**Authors:** Mitsunori Ikeda, Toshihiro Kitai, Nobuhiro Hayashi, Isao Ukai, Toshikatsu Nobunaga, Masanobu Kohno, Tatsuya Sugino

**Affiliations:** 1 Department of Emergency and Critical Care Center, Hyogo Prefectural Nishinomiya Hospital, 13-9 Rokutanji-cho, Nishinomiya, Hyogo 662-0918 Japan; 2 Department of Obstetrics and Gynecology, Hyogo Prefectural Nishinomiya Hospital, 13-9 Rokutanji-cho, Nishinomiya, Hyogo 662-0918 Japan

**Keywords:** Colonic ischemia, Amniotic fluid embolism, Resuscitative endovascular balloon occlusion of the aorta

## Abstract

**Background:**

Resuscitative endovascular balloon occlusion of the aorta (REBOA) can control massive postpartum hemorrhage.

**Case presentation:**

A 41-year-old woman transferred to hospital following cesarean section presented in refractory hemorrhagic shock. REBOA was blindly performed in the emergency department. She immediately underwent hysterectomy and damage control surgery in the operating room. The aortic balloon, whose position was confirmed at zone II by postoperative X-ray, provided intermittent occlusion for 40 min during surgery. Hemodynamics were stabilized with these interventions, with massive transfusion required for severe coagulopathy perioperatively. She gradually recovered with intensive care but suffered ascending colon ischemia with perforation on day 16. She received a colostomy and was discharged without sequelae after 130 days. Amniotic fluid embolism was diagnosed according to clinical criteria and supplemental serum markers.

**Conclusions:**

This patient suffered colonic ischemia possibly due to REBOA used to manage amniotic fluid embolism. REBOA requires careful consideration to avoid complications.

## Background

Amniotic fluid embolism (AFE) is a rare but potentially fatal complication of pregnancy. Massive exsanguination followed by severe coagulopathy is a critical occurrence in patients with AFE. Recently, resuscitative endovascular balloon occlusion of the aorta (REBOA) is being considered a feasible and safe alternative tool for postpartum hemorrhage [1]. We report a case of colonic ischemia possibly caused by REBOA used in the management of AFE.

## Case presentation

**Fig. 1:**
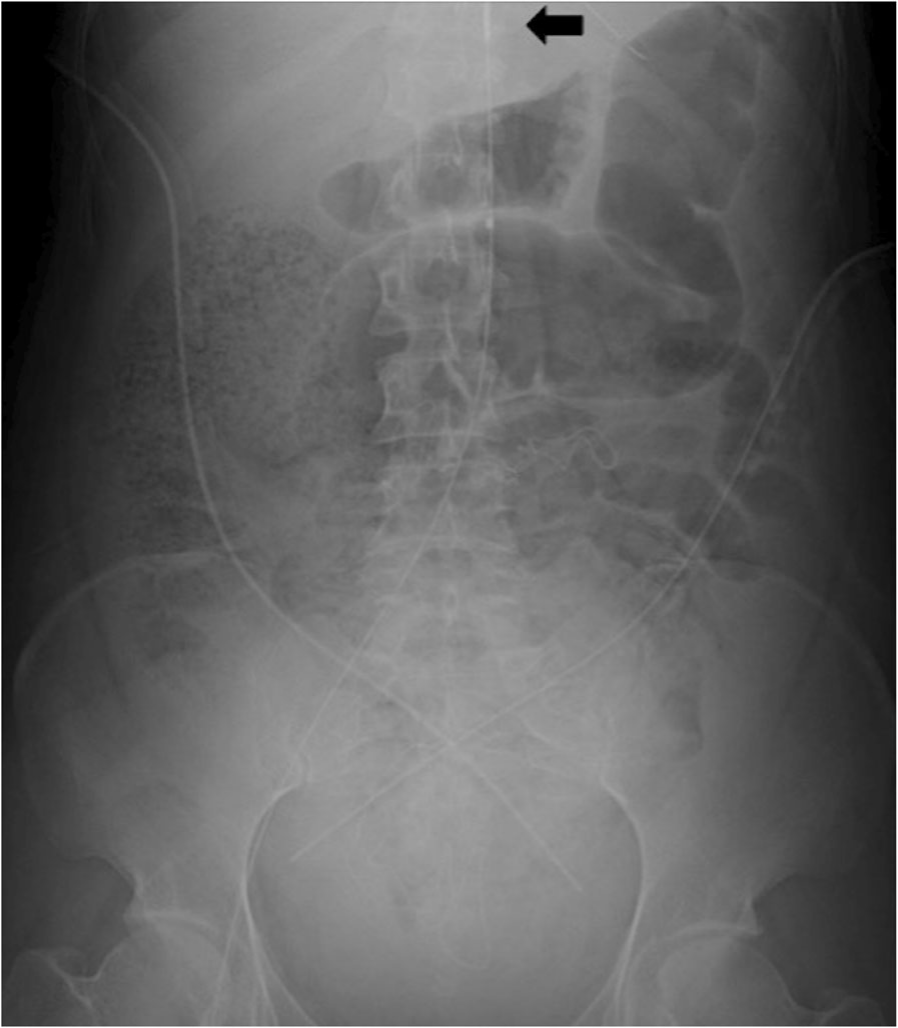
Balloon position was confirmed by abdominal X-ray in the operating room after surgery. The black arrow indicates the tip of the catheter placed at zone II of the aorta

A 41-year-old woman, gravida 5 para 3, at 41 weeks of gestation underwent emergency cesarean section at her regular clinic because of fetal distress following induction of labor. Soon after delivery, she developed hemodynamic instability with genital bleeding and was transferred to our hospital. On arrival, she presented in refractory shock with the following vital signs: Glasgow coma scale, E2V4M5; respiratory rate, 28 breaths/min; heart rate, 140 bpm; systolic blood pressure, < 60 mmHg and SpO_2_, 98% (O_2_, 10 L/min). Blood gas analysis showed pH 6.879, PaCO_2_ 33 mmHg, PaO_2_ 461 mmHg, HCO_3_^−^ 5.8 mmol/L, lactate 13.9 mmol/L, and hemoglobin 3.9 g/dL. Coagulation and fibrinolysis testing revealed values of 25 mg/dL for fibrinogen, 17% for prothrombin activity, 151 μg/mL for D-dimer, and 21% for antithrombin III activity. The patient was endotracheally intubated, and massive transfusion was initiated along with clamping of the lateral ligament of the uterus. Despite these interventions, her hemodynamic instability persisted. Therefore, we decided to initiate REBOA to prevent cardiac arrest. We accessed the right femoral artery by Seldinger technique and aimed to place the balloon at aortic zone III. We deployed the balloon blindly based on manual estimation and inflated the balloon. Hemodynamic stability was temporarily obtained with a systolic blood pressure of > 100 mmHg. She was immediately transferred to the operating room, and hysterectomy was performed to definitively control the hemorrhage. Diffuse bleeding persisted around the surgical field as a result of severe coagulopathy, and therefore, temporary abdominal closure was subsequently performed with peritoneal gauze packing as a damage control procedure. The REBOA balloon was gradually deflated with careful hemodynamic monitoring during surgery, and total occlusion time was 40 min. Duration of the operation was 78 min, and blood loss was over 5000 mL. After surgery, an abdominal X-ray performed in the operating room revealed that the balloon was positioned in zone II of the aorta (Fig. 1). In the perioperative setting, the patient required 130 units of red blood cells, 130 units of fresh frozen plasma, and 260 units of platelets, and recombinant-activated factor VIIa (rFVIIa) was also administered to control hemorrhage.

**Fig. 2:**
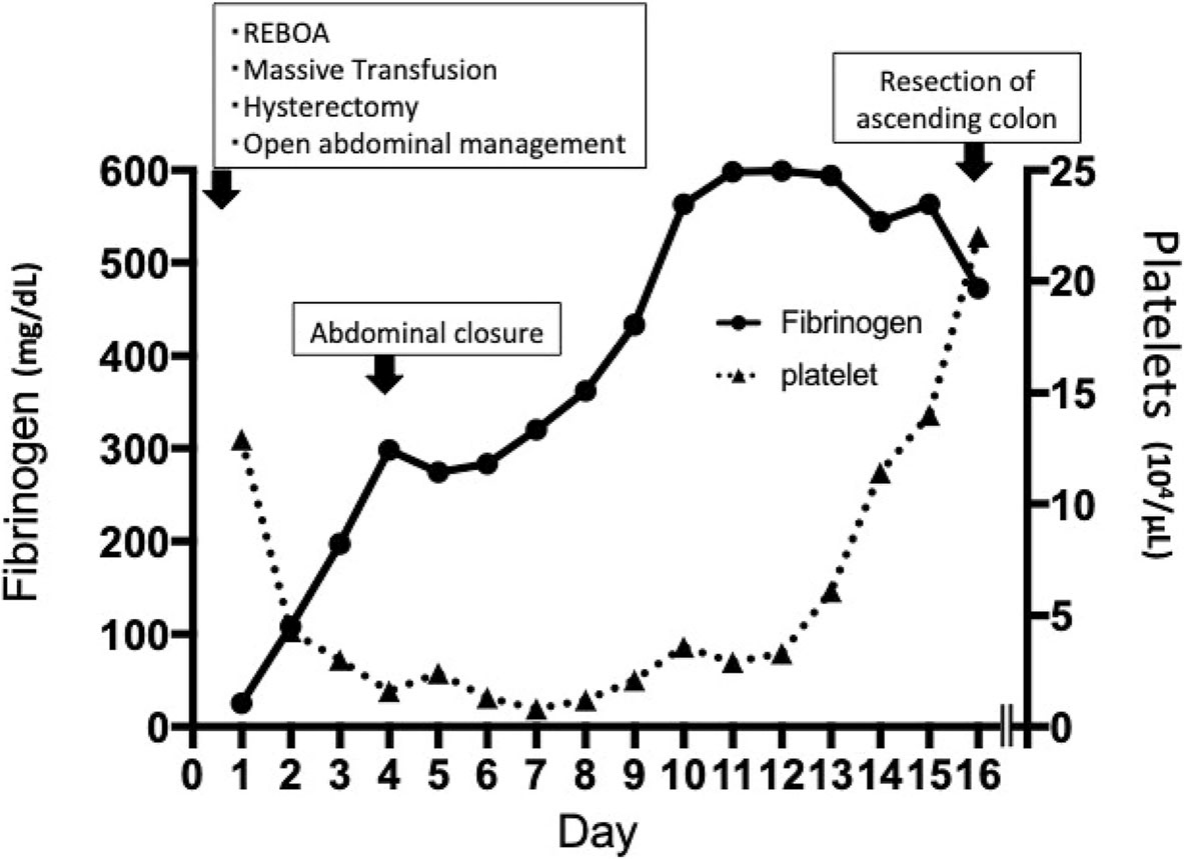
Time course of platelet counts and fibrinogen level. REBOA resuscitative endovascular balloon occlusion of the aorta

The patient underwent typical abdominal closure on hospital day 4 and was extubated on day 14. We diagnosed AFE according to the clinical features and results of supplemental serum markers such as zinc coproporphyrin-1 (8.9 p mol/mL), sialyl Tn (52 U/mL), C3 (46 mg/dL), C4 (5.0 mg/dL), and C1 esterase inhibitor (< 25%). Although her condition gradually improved with intensive care, she suffered massive bleeding from the lower digestive tract on day 16. An abdominal computed tomography scan showed necrosis of the ascending colon with perforation, for which a colostomy was immediately performed (Fig. 2). She recovered from septic shock due to peritonitis and was moved to the general ward after a 47-day stay in the ICU. She was discharged home without any sequelae after a 130-day hospital stay.

## Discussion

AFE is a rare but life-threatening crisis with a high mortality rate of 20–40% in pregnant women [2]. The pathophysiological mechanism has not been adequately clarified, but it is hypothesized that entrance of some materials derived from fetal components into the maternal circulation induces activation of maternal systemic inflammatory responses, resulting in pulmonary vasoconstriction, acute heart failure, and disseminated intravascular coagulation [3].

Most patients with AFE progress rapidly to cardiovascular collapse with massive bleeding from surgical fields, which may lead to cardiac arrest. Therefore, the treatment strategy to control hemorrhage is important in the management of AFE.

Recently, an increasing number of reports have shown the effectiveness of REBOA in controlling major exsanguinating hemorrhage. REBOA has been used as an alternative application for proximal control of massive hemorrhage in patients with trauma or ruptured aortic aneurysm [4], and the experience with REBOA for postpartum hemorrhage has been reported in several articles [1]. REBOA is widely considered to be a minimally invasive and safe technique. A recent systematic review reported a rate of iatrogenic injury related to REBOA of < 5% [5]. Meanwhile, the potential risks of REBOA have also been indicated. Sadeghi et al. reported that several complications such as balloon migration (4%), balloon rupture (3%), and extremity compartment syndrome (7%) were observed, even though no major complications including access site bleeding or aortic injury occurred [6]. Furthermore, a previous study reported the adverse events of distal ischemia and reperfusion injury, such as lower limb ischemia and acute kidney injury [7]. The present patient suffered the rare but serious complication of colonic ischemia with perforation possibly due to REBOA.

There are several considerations with regard to this complication. First, the balloon was inflated in aortic zone II. Occlusion positions were defined to provide a comprehensive understanding for the use of REBOA [8]. Zone I extends from the origin of the left subclavian artery to the celiac artery, which makes it theoretically feasible for the proximal control of hemorrhage. Zone II is the paravisceral segment, which includes branches such as the celiac artery, mesenteric artery, and renal artery. Zone II is generally proposed as an area of non-occlusion because occlusion would be potentially incomplete due to these branches. Zone III extends from the lowest renal artery to the aortic bifurcation, which is suitable for control of pelvic or postpartum bleeding. In the present case, the aortic balloon was blindly inserted in the emergency room and consequently delivered to zone II unexpectedly. This location might have led to the ascending colon ischemia due to partial occlusion of the superior mesenteric artery. A previous study indicated that the success rate in deploying the balloon in the correct location was not necessarily high, and adjustment of the position was made in 34% of cases [6]. The REBOA position in our patient was not confirmed by imaging until after surgery because we made a decision at the moment that preferred hysterectomy to control the hemorrhage. If the balloon had been appropriately located in zone I or zone III, we potentially could have avoided the ischemic complication. Physicians need to keep in mind that the occlusion position needs to be confirmed as soon as possible via portable radiography or ultrasound. Second, we inflated the balloon intermittently and partially for a total of 40 min in our patient. Several studies indicated that an optimal occlusion time of 20 or 30 min is recommended because of the risks of organ failure due to ischemia-reperfusion injury [9]. It is worth noting that the aortic balloon must be deflated immediately after hemorrhagic control is obtained. Third, it was unclear who was responsible for the management of REBOA. From a technical point of view, REBOA was performed by an emergency physician with adequate experience and skill. However, the primary physician simultaneously acted as the leader during the treatment of the patient. A multidisciplinary approach could potentially help to reduce complications of REBOA. Finally, rFVIIa was given to control the diffuse hemorrhage after surgery. Some previous studies proposed the effectiveness of rFVIIa in AFE cases [10]. However, Leighton et al. suggested that rFVIIa would be detrimental for AFE patients because rFVIIa triggers a thrombin burst, which may lead to increased thrombotic complications [11]. Although there was no evidence of a direct relation between rFVIIa and the ischemic change in the colon of our patient, the use of rFVIIa for AFE patients should be limited to cases in which first-line therapy fails to control bleeding. In conclusion, we reported a case of colonic ischemia possibly due to REBOA used in the management of AFE. The potential risks of REBOA should be clear in this case. Careful consideration is required in the management of REBOA.

## Data Availability

Not applicable.

## Abbreviations

AFE: Amniotic fluid embolism
REBOA: Resuscitative endovascular balloon occlusion of the aorta
rFVIIa: Recombinant activated factor VIIa
